# Ethnobotanical Evidence of Medicinal Plants Used for Peptic Ulcers in Tanzania: A Systematic Review

**DOI:** 10.1155/sci5/2126859

**Published:** 2026-05-24

**Authors:** David Sylvester Kacholi

**Affiliations:** ^1^ Department of Biological Sciences, Dar es Salaam University College of Education, University of Dar es Salaam, P.O. Box 2329, Dar es Salaam, Tanzania, udsm.ac.tz

**Keywords:** antiulcer, duodenal ulcer, ethnomedicine, *Helicobacter pylori*, phytomedicine, stomach ulcer

## Abstract

Medicinal plants (MPs) are essential to rural communities in low‐ and middle‐income countries. However, knowledge of the use of MPs for peptic ulcer disease (PUD) in Tanzania remains limited. This review aims to document the traditional knowledge and MPs used by Tanzanians to treat and manage PUD. A comprehensive search was conducted across databases and grey literature, adhering to the Preferred Reporting Items for Systematic Reviews and Meta‐Analyses (PRISMA) guidelines. Data from 26 ethnobotanical studies were analysed using descriptive statistics (means, graphs and charts) in Microsoft Excel. Overall, 101 MPs from 44 botanical families have been traditionally used to treat and manage PUD in Tanzania. Asteraceae (10.8%) and Fabaceae (9.6%) were the most diverse families. In terms of life forms, trees account for 40% of the documented plants, followed by herbs at 39%. The most commonly used plant parts are leaves (40%) and roots (32%). Decoction (58%) is the most common method for preparing these remedies, with oral administration as the primary route. The most frequently cited MPs include *Sclerocarya birrea* (A. Rich.) Hochst. (Anacardiaceae), *Dichrocephala integrifolia* (L.f.). Kuntze (Asteraceae), *Ageratum conyzoides* L. (Asteraceae), *Dodonaea viscosa* Jacq. (Sapindaceae) and *Cissus rotundifolia* Lam. (Vitaceae). Of the recorded MPs, 32 have been validated for antiulcerogenic activity and should advance to standardised formulations, dosage optimisation and clinical trials to enable integration into modern, evidence‐based healthcare. Conversely, this study recommends that unvalidated MPs be systematically studied for efficacy and safety to ensure that culturally rooted remedies are evidence‐based and safely integrated into contemporary healthcare.

## 1. Introduction

Globally, over 80% of the population relies on traditional medicine for health concerns [1]. Herbal products have played a significant role in identifying numerous bioactive compounds since antiquity. Herbal products, long recognised for bioactive compounds, remain central to managing chronic diseases, including peptic ulcer disease (PUD). They are often perceived as safer than synthetic pharmaceuticals and are increasingly explored as alternatives for long‐term conditions, such as ulcers, diabetes, cancer and cardiovascular disorders [2–5]. Recent studies have substantiated the safety and efficacy of plant‐based remedies for treating PUD and other medical conditions [6, 7].

PUD is a chronic gastrointestinal disorder marked by acid‐induced gastric and duodenal lesions, contributing to significant morbidity and mortality [8]. Symptoms include epigastric pain, nausea, heartburn, bloating, vomiting, bleeding and weight loss [9–12]. Globally, the prevalence is estimated at 156.6 per 100,000, with higher rates in low‐ and middle‐income countries [8, 13]. In Africa, regional variation is evident: West Africa reports the highest prevalence (19%), followed by East (15%), North (12%) and Southern Africa (8%). Among nations, Ghana (27%), Ethiopia (19%) and Tanzania (16%) record the highest burdens [13]. PUD affects both genders equally and may occur at any age, with a notable onset between 10 and 15 years [2, 13, 14]. Unlike other chronic conditions, PUD is marked by acute, potentially fatal complications such as gastrointestinal bleeding and perforation [15]. Its combination of persistent symptoms and sudden emergencies elevates its importance for clinical care and public health policy [8, 16].

An imbalance between defensive factors, including reduced bicarbonate, nitric oxide (NO), prostaglandins (PGs), antioxidants and aggressive factors, such as free radicals, excessive gastric acid and pepsin, represents a crucial mechanism in the pathogenesis of the disease. Recent studies have indicated that the risk factors associated with PUD globally include *Helicobacter pylori* infection, the usage of aspirin, Zollinger–Ellison syndrome, drinking of alcohol and nonsteroidal anti‐inflammatory drugs (NSAIDs) [11, 17]. Management of chronic PUD depends on the ulcer’s cause, particularly its association with *H. pylori* or NSAIDs, the ulcer’s status (new or recurrent) and any complications. The main goal of treatment is to minimise complications, promote ulcer healing and prevent recurrence. For patients who are positive for *H. pylori* and have an active ulcer or a history of complications, treatment focuses on eradicating *H. pylori* and promoting ulcer healing to achieve definitive resolution [11, 18, 19].

PUD remains a significant health burden in Tanzania, with studies reporting high prevalence among dyspeptic patients and a persistent association with *Helicobacter pylori* infection [20]. Despite global declines in morbidity, limited access to endoscopy, eradication therapies and diagnostic resources continues to exacerbate the burden locally [21, 22]. These constraints highlight the urgent need for targeted research and interventions in low‐resource settings. Ethnobotanical research in Tanzania has documented the use of medicinal plants (MPs) for a wide range of ailments, including PUD, yet most studies emphasise broad therapeutic applications rather than disease‐specific treatments. The lack of systematic documentation of MPs used for PUD underscores the need for comprehensive ethnobotanical data to inform both clinical practice and public health strategies. Therefore, this review aims to document the traditional practices employed by Tanzanians in treating or managing PUD by addressing the following questions: (1) Which MPs do various ethnic groups use for PUD? (2) What are the ethnic and regional distributions of these MPs? (3) Which parts of MPs are used? (4) How are MPs prepared to treat PUD? (5) What is the route of administration for the remedies?

## 2. Methodology

**FIGURE 1:**
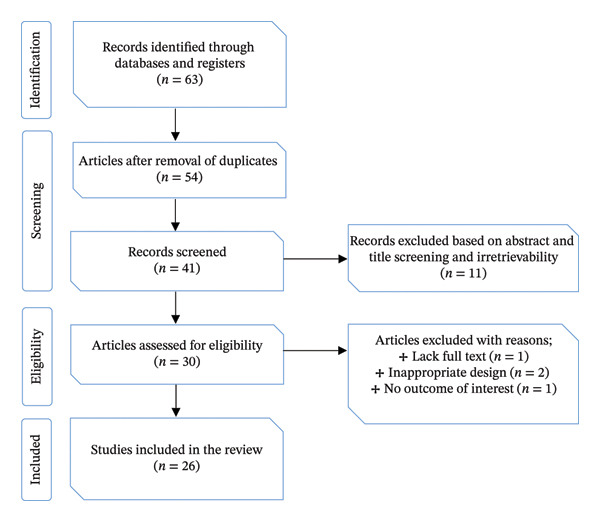
PRISMA flow diagram showing the number of studies through different phases of the systematic review.

This review critically examines the use of herbal medicines traditionally applied in the management of PUD in Tanzania, integrating evidence from a wide range of sources. Between May 2024 and March 2025, a systematic compilation of indigenous medical practices was undertaken, complemented by an analysis of conventional knowledge across diverse ethnic groups. The review was conducted with methodological rigour, adhering to the Preferred Reporting Items for Systematic Reviews and Meta‐Analyses (PRISMA) guidelines (Figure 1).

### 2.1. Literature Search Strategy

Ethnobotanical studies documenting MPs for PUD management in Tanzania were identified through databases such as Scopus, PubMed, Wiley Online Library, Google Scholar, African Journals Online (AJOL) and Web of Science. Relevant websites and doctoral dissertations were also explored using Google search. The key terms employed during the search were ‘peptic ulcers’, ‘ulcers’, ‘gastric ulcers’, ‘duodenal ulcers’, ‘traditional medicinal plants’, ‘medicinal plants’, ‘traditional knowledge’, ‘indigenous knowledge’, ‘ethnobotany’, ‘ethnobotanical study’, ‘ethnomedicine’, ‘herbal remedies’, ‘United Republic of Tanzania’ and ‘Tanzania’. The search terms were used separately and, at times, combined with Boolean operators such as ‘OR’ or ‘AND’.

### 2.2. Inclusion Criteria

The inclusion criteria encompassed both published and unpublished ethnobotanical studies reporting MPs associated with PUD in Tanzania. The review was restricted to original research articles written in English. Articles were considered eligible if they documented the botanical name, local name, plant parts utilised, methods of preparation and routes of administration of medication. Verification of botanical nomenclature, along with supplementation of missing details such as growth habits and life forms, was conducted using the Plant of the World Online database (https://powo.science.kew.org/).

### 2.3. Exclusion Criteria

Studies lacking essential ethnobotanical information, such as scientific names, plant parts used, routes of administration or study locations, were excluded from the analysis. Likewise, research focusing solely on MPs for livestock, publications with restricted accessibility, abstracts without full texts, non–open‐access sources, studies conducted outside Tanzania and articles not published in English were omitted.

### 2.4. Study Selection

The screening of studies was conducted in two sequential stages to ensure methodological rigour. First, titles and abstracts of all retrieved records were examined to determine preliminary eligibility. Articles that appeared relevant were then subjected to full‐text review, during which the predefined inclusion and exclusion criteria were systematically applied. This two‐step process minimised the risk of bias and ensured that only studies meeting the established methodological standards were incorporated into the final analysis. Ultimately, the review comprised 26 studies that satisfied all eligibility requirements and were included in the analysis (Figure 1).

### 2.5. Data Analysis

Descriptive statistical methods were employed to analyse and summarise the data using MS Excel software. The results are reported as percentages and frequencies, and are systematically presented through tables, bar charts and pie charts to facilitate rigorous interpretation and comparison.

### 2.6. Study Limitations

Limitations of this review include the lack of protocol registration (e.g., PROSPERO), risk‐of‐bias assessment and study quality appraisal. Keyword overlap across databases led to data duplication, reducing search completeness. Furthermore, article screening and cross‐checking were conducted by a single author, which may introduce bias compared to multireviewer systematic reviews.

## 3. Results and Discussion

Usually, ethnobotanical studies require standard procedures for botanical identification and documentation of indigenous knowledge related to plant distribution, management and traditional medicinal use. A total of 26 ethnobotanical studies on MPs used for the treatment of PUD in Tanzania were retrieved from different electronic databases.

### 3.1. Distribution of MPs

**FIGURE 2:**
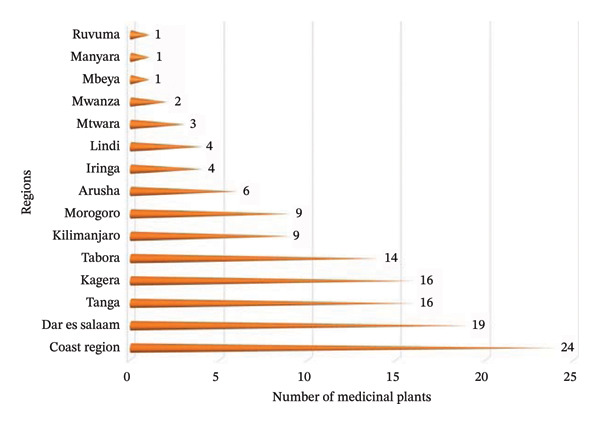
Regional distribution of antiulcer medicinal plants in Tanzania.

This review has thoroughly examined 25 ethnobotanical studies conducted across 15 of the 31 administrative regions in Tanzania. The findings reveal a significant gap, highlighting that research on traditional remedies for PUD across the country remains limited. Notably, the Coast region stands out as the most documented area, boasting an impressive 24 MPs known for their antiulcer properties. Following closely are the regions of Dar es Salaam (19 MPs), Tanga (16 MPs), Kagera (16 MPs), Tabora (14 MPs), Kilimanjaro (9 MPs), Morogoro (9 MPs) and Arusha (6 MPs). The remaining regions report fewer than 5 MPs, underscoring the disparity in ethnomedicinal exploration across the country (Figure 2). The abundance of reported antiulcer plants in these particular regions suggests that local communities possess a rich repository of indigenous knowledge regarding the utilisation of these MPs. Therefore, the observed trend may indicate a comparatively higher prevalence of PUD in these regions, potentially attributable to the interplay between prevailing cultural practices and broader health challenges faced by the population.

### 3.2. Diversity of MPs and Indigenous Knowledge

A total of 101 MPs from 44 botanical families were identified for the treatment and management of PUD, based on an examination of 25 reviewed articles (Table 1). This review provides substantial evidence of individuals’ extensive indigenous knowledge, demonstrating how they continue to utilise traditional methods for treating and managing PUD within the country. Asteraceae comprising 9 species (10.8%) were the most highly represented families, followed by Fabaceae (8 species, 9.6%), Annonaceae and Lamiaceae (each with 5 species, 6.0%) and Amaranthaceae, Anacardiaceae and Cucurbitaceae (each with 4 species, 4.8%) (Table 1). These findings align with other reviews [49–51], indicating that Asteraceae and Fabaceae are the most represented families. This finding is also consistent with a study conducted in Ethiopia, in which Asteraceae and Fabaceae were the most commonly used in plant‐based rituals for the prevention and treatment of PUD [52]. The phytochemical constituents frequently reported in members of well‐represented families encompass, but are not restricted to, tannins, triterpenes, phenols, flavonoids, saponins, terpenoids, alkaloids and glycosides, which are categorised as secondary metabolites that help protect the gastric lining and reduce inflammation [53–56]. Therefore, this review suggests that further research is required to ascertain the extent to which these secondary metabolites contribute to the management of PUD.

**TABLE 1:** Medicinal plants used to treat and manage peptic ulcer disease in Tanzania.

Family	Species name	Vernacular name (tribe)	LF	PU	Preparation and administration	CS	Reference
Acanthaceae	*Pseudospondias microcarpa* (A. Rich) Engl.	Omuzilu/Mziku (Haya)	T	B, Fr	Decoction drunk	LC	[23]
	*Thunbergia alata* Bojer ex Sims	Nakagwinda (Ngindo)	H	L	Decoction drunk	—	[24, 25]

Amaranthaceae	*Amaranthus spinosus* L.	Olulele (Haya)	H	L	Boiled with unskimmed cow milk and then drunk	—	[26]
	*Chenopodium opulifolium* Schrad. ex W.D.J.Koch & Ziz	Omwetango (Haya)	H	L	Decoction drunk	—	[26]
	*Dysphania ambrosioides* (L.) Mosyakin & Clemants	Mdiwasoko (Chagga)	H	L	Not specified	—	[27]
	*Pupalia lappacea* (L.) Juss.	Mnamata (Kwere/Swahili)	H	Fr	Not specified	LC	[28]

Anacardiaceae	*Ozoroa insignis* Delile	Omukerenge (Haya)	T	Sb, R	Root or bark powder is mixed with black tea and taken orally	LC	[29]
	*Ozoroa mucronata* (Bernh.) R.Fern. & A.Fern.	Muungwae (Ndengereko), Mgombo kilangu (Zaramo), Mkalakala (Zigua), Mlago (Sukuma)	T	L	Pounded leaves rubbed on the stomach	LC	[24]
	*Schinus molle* L.	Mpilipili (Luguru)	T	R	Not specified	LC	[30]
	*Sclerocarya birrea* (A. Rich.) Hochst.	Ormangwai/olmang’oi. (Maasai), Mgoto (Kwere), Mng’ongo (Swahili)	T	R, B	Not specified	LC	[28, 31, 32]

Annonaceae	*Annona senegalensis* Pers.	Mbokwe (Sambaa), Mtopetope (Swahili)	T	R	Not specified	LC	[33]
	*Hexalobus monopetalus* (A.Rich.) Engl. & Diels	Mkuwa (Nyamwezi)	T	L	Decoction drunk	LC	[34]
	*Uvaria sofa* Scott Elliot	Msofa (Kwere/Swahili)	S	L	Not specified	LC	[28]
	*Xylopia longipetala* De Wild. & T.Durand	Mlawilila (Luguru)	T	B	Not specified	LC	[35]
	*Xylopia odoratissima* Welw. ex Oliv.	Mshenene (Nyamwezi)	T	L, R	Decoction drunk	LC	[34, 36]

Apiaceae	*Choritaenia capensis* Benth.	Mhungulu (Hehe)	H	L	Not specified	—	[30]
	*Daucus carota* L.	Ekaroti (Haya)	H	Fr	Eaten raw	—	[29]

Asphodelaceae	*Aloe barbadensis* Miller	Enkaka	Su	L	Leaf extract mixed with honey and raw eggs; one tablespoon taken orally	—	[29]
	*Aloe vera* (L.) Burm. f.	Mlovera (Swahili)	H	Whp	Decoction drunk	—	[34]

Asteraceae	*Ageratum conyzoides* L.	Omwigara (Haya)	H	R, L	Roots are chewed fresh as an antacid, while the leaves’ decoction is drunk	LC	[23, 26, 37]
	*Bidens pilosa* L.		H	L	Not specified	—	[38]
	*Crassocephalum vitellinum* (Benth.) S.Moore	Ekishenda (Haya)	H	Ap	Decoction drunk	—	[23]
	*Dichrocephala integrifolia* (L.f.) Kuntze	Shinda kaya (Sambaa), Ibuza (Haya)	H	L	Decoction drunk	—	[26, 33, 37]
	*Emilia coccinea* (Sims) G. Don	Kanyoro (Haya)	H	Whp	Decoction drunk		[29]
	*Lipotriche scandens* (Schumach. & Thonn.) Orchard	Byabarwoya (Haya)	H	L	Decoction drunk	—	[26, 37]
	*Solanecio angulatus* (Vahl) C.Jeffrey	Leza (Sambaa)	S	L	Not specified	LC	[38]
	*Solanecio cydoniifolius* (O.Hoffm.) C.Jeffrey	Hanamwiko (Luguru).	H	L	Infusion drunk	—	[27]
	*Sonchus oleraceus* L.	Orunoko (Haya)	H	Whp	Decoction drunk	—	[29]

Bignoniaceae	*Stereospermum kunthianum* Cham.	Mkomanguku (Zaramo)	T	L	Decoction drunk	LC	[24]
	*Kigelia africana* (Lam.) Benth.	Mwegea (Sambaa)	T	R	Not specified	LC	[33]

Brassicaceae	*Brassica oleracea* var. capitata	Ekabeji (Haya)	H	Whp	Squeezing to make juice then drunk	—	[29]

Burseraceae	*Commiphora africana* (Rich.) Engl.	Silalei, Tanguta (Chagga, Nyakyusa, Luguru, Pogoro)	T	Fr	Not specified	LC	[31]
	*Commiphora swynnertonii* Burtt	Oltemwai (Maasai)	S	B	Not specified	—	[32]

Caricaceae	*Carica papaya* L.	Mpapai (Hehe)	T	L	Not specified	DD	[30]

Celastraceae	*Gymnosporia senegalensis* (Lam.) Loes.	Omunyaburiko (Haya)	S	R	Powdered leaves mixed with black tea and drunk	LC	[29]

Cleomaceae	*Cleome viscosa* L.	Mgagani (Swahili)	H	R, L, B	Not specified	—	[32]

Combretaceae	*Combretum zeyheri* Sond.	Musana (Nyamwezi)	S	R, L, B	Chew and swallow the juice	LC	[36]
	*Terminalia sambesiaca* Engl. & Diels	Mpuku (Pare), Msame‐dume (Hehe).	T	B, L	Decoction drunk	LC	[27]
	*Terminalia sericea* Burch.ex DC.	Muzima (Nyamwezi)	T	R	Infusion drunk	LC	[34]

Convolvulaceae	*Bonamia mossambicensis* (Klotzsch) Hallier f.	Mlipu (Kwere/Swahili)	H	L	Not specified	—	[28]

Cucurbitaceae	*Cucumis ficifolius* A.Rich.	Sharahandid (Barbaig)	H	Fr	Pounded and juice drunk	—	[39]
	*Cucumis sativus*	Amatango (Haya)	Cl	Fr	Eaten raw	—	[29]
	*Cucurbita pepo*	Omwongo (Haya)	Cl	Se	Seeds roasted and taken orally	—	[29]
	*Lagenaria sphaerica* (Sond.) Naudin	Entanga (Haya)	Cl	Se	Roasted and eaten	—	[29]

Ebenaceae	*Euclea natalensis* A.DC.	Mlamamwitu (Swahili), Mnindimya (Matumbi), Mdala (Zigua),	T	R	Decoction drunk	LC	[27]
	*Euclea divinorum* Hiern	Mdaa (Swahili)	S	L	Not specified	LC	[40]

Euphorbiaceae	*Jatropha curcas* L.	Ekiho (Haya)	T	Sb	Decoction drunk	LC	[29]
	*Ricinus communis* L.	Legezabwende (Vidunda) Mbarika, Mbono (Swahili).	T	L	Not specified	LC	[41]
	*Tragia furialis* Bojer	Omugonampili (Haya)	T	L	Decocted with milk and then drunk	—	[26]

Fabaceae	*Abrus precatorius* L.	Mwangaruchi, Luvambo (Pare)	H	Se	Not specified	—	[42]
	*Alantsilodendron pilosum* Villiers	Chikula gembe (Sambaa), Mgegele (Hehe)	T	R	Infusion drunk	LC	[30, 43]
	*Arachis hypogaea* L.	Karanga (Swahili), Ekinyobwa (Haya)	H	Se	Decoction drunk or powder mixed with honey and taken orally thrice a day	—	[29, 34]
	*Cassia abbreviata* Oliv.	Mwalola (Makonde)	T	B	Powdered bark then mixed in water and drunk	LC	[33]
	*Indigofera arrecta* Hochst. ex A.Rich.	Emuchukuchuku (Maasai)	H	R, L	Not specified	—	[44]
	*Neorautanenia ficifolia* (Benth. ex Harv.) C.A.Sm	Tuha (Sambaa)	H	R	Roots burned and ashes applied externally to the stomach through rubbing	—	[45]
	*Piliostigma thonningii* (Schumach.) Milne‐Redh.	Msegese (Swahili)	T	L	Decoction drunk	LC	[24]
	*Tamarindus indica* L.	Mkwedu (Makonde), Mkwaju (Swahili)	T	Fr	Not specified	LC	[33]

Lamiaceae	*Coleus barbatus* (Andrews) Benth. ex G.Don	Mbelasigulu (Nyamwezi)	H	R	Infusion mixed with honey, then drunk	—	[34]
	*Leonotis nepetifolia* (L.) R.Br.	Igicumucumu (Hangaza)	H	Whp	Decoction drunk	—	[46]
	*Premna chrysoclada* (Bojer) Gürke	Mvuma (Zigua), Mjavikari, Makarikana (Masai)	S	L	Not specified	LC	[42]
	*Tetradenia riparia* (Hochst.) Codd	Mkono wa nkhanda, kiswija, Omushunshu (Haya)	H	L	Decoction drunk	LC	[23]
	*Tetradenia urticifolia* (Baker) Phillipson	Omushunshu (Haya)	H	L	Decoction drunk	—	[26]

Lauraceae	*Cassytha filiformis* L.	Hauna shina (Zaramo), Mlangamia (Zigua)	H	Whp	Not specified	—	[41]
	*Cinnamomum verum* J.Presl	Omudalasini (Haya)	T	Sb	Powder mixed with honey then drunk	—	[29]
	*Persea americana* Mill.	Ekivakedo (Haya)	T	Se	Powdered seeds are mixed with black tea and taken orally	LC	[29]

Loganiaceae	*Strychnos innocua* Del.	Mpundu (Nyamwezi)	H	B	Decoction drunk	LC	[34]

Loranthaceae	*Plicosepalus sagittifolius* (Engl.) Danser	Kimpa eha mkutani (Pare)	H	L	Squeeze and the juice drunk	—	[41]

Lythraceae	*Punica granatum* L.	Mkomamanga (Swahili)	T	R, Fr	Decoction drunk	LC	[28, 34]

Meliaceae	*Trichilia emetica* Vahl	Mrikawandu (Chagga).	T	B	Not specified	LC	[41]
	*Khaya nyasica* Stapf ex Baker f.	Mkangazi (Swahili)	T	R	Not specified	VU	[32]

Moraceae	*Ficus asperifolia* Miq.	Ekijuhuju/Omuku (Haya)	S	L	Decoction drunk	LC	[23]
	*Ficus exasperata* Vahl.	Omusomolo (Haya)	T	L	Leaves burned and ashes taken orally	LC	[29]
	*Ficus lutea* Vahl.	Mvule (Swahili)	T	B	Infusion drunk	LC	[43]

Moringaceae	*Moringa oleifera* Lam.	Mlonge (Swahili), Omulonge (Haya)	T	Fr	Infusion drunk	LC	[29, 34]

Musaceae	*Musa acuminata* Colla	Ekitooke (Haya)	T	Fr	Dried ripe and unripe bananas are powdered, mixed with honey and taken orally	LC	[29]
	*Musa sapientum* L.	Mugomba (Nyamwezi)	H	Fr	Peel the fruit, then eat it as food	LC	[34]

Myricaceae	*Myrica salicifolia* Hochst. ex A.Rich.	Olkitalaswa (Maasai), Omukikimbo (Haya)	S	L, R	Decoction drunk	LC	[29, 44]

Myrtaceae	*Psidium guajava* L.	Mpera (Swahili)	T	B, L	Not specified	LC	[30, 32]
	*Syzygium cumini* (L.) Skeels	Musambarao (Nyamwezi)	T	L	Infusion drunk	LC	[34]

Nyctaginaceae	*Boerhavia coccinea* Mill.	Kimotoka (Haya)	H	Ap	Infusion drunk	—	[37]

Ochnaceae	*Ochna atropurpurea* DC.	Mkeremange (Zigua)	S	Whp	Not specified	—	[45]

Olacaceae	*Ximenia afra* Sond.	Mpingi (Swahili), Mtundwi (Zigua), Mtundutwa (Pare), Mnembwa (Nyamwezi)	T	R, L	Decoction mixed with beans and peas is drunk	LC	[36, 45]
	*Ximenia americana* L.	Lusasalwake (Zigua), Mtundwa (Sukuma).	T	R	Not specified	LC	[45]

Phyllanthaceae	*Antidesma membranaceum* Müll.Arg.	Iramba, Ikararata (Pare), Ngetzi (Chagga)	T	R	Not specified	LC	[47]
	*Antidesma venosum* E.Mey. ex Tul.	Enjanemeno (Maasai), Mkandekande (Swahili)	S	R, B	Not specified	LC	[31, 32]
	*Phyllanthus amarus* Schumach. & Thonn.	Mturuka (Swahili)	H	L	Not specified	—	[31]

Plumbaginaceae	*Plumbago zeylanica* L.	Enkira/Enkila (Haya)	S	Ap	Decoction drunk	—	[23]

Poaceae	*Sorghum bicolor* (L.) Moench	Mtama (Swahili)	H	Se	Not specified	LC	[28]

Polygonaceae	*Oxygonum sinuatum* (Hochst. & Steud. ex Meisn.) Dammer	Akachumitambogo (Haya)	H	Whp	Decoction drunk	—	[29]
	*Securidaca longepedunculata* Fresen.	Muteyu (Nyamwezi)	S	R	Powder mixed with hot water and drunk	LC	[34]

Rhizophoraceae	*Cassipourea mollis* (R.E. Fries) Alston	Mlugala (Nyamwezi)	T	R	Chew, then swallow the fluid	LC	[34]

Rubiaceae	*Crossopteryx febrifuga* (Afzel. ex G.Don) Benth.	Nakapwendo (Makonde), Nkolokolo (Yao), Msikosiko (Zigua)	T	R	Not specified	LC	[42, 48]
	*Rutidea orientalis* Bridson	Mtitu (Luguru)	S	R	Not specified	LC	[30]
	*Vangueria infausta* Burch.	Msada, Mvilu (Zigua).	T	R	Decoction drunk	LC	[48]

Rutaceae	*Zanthoxylum chalybeum* Engl.	Mlungulungu (Swahili, Nyamwezi), Mhunungu (Luguru)	S	R	Decoction drunk	LC	[30, 34]

Salicaceae	*Flacourtia indica* (Burm.f.) Merr.	Mbuguswa (Pogoro)	S	R	Not specified	LC	[31]

Sapindaceae	*Dodonaea viscosa* Jacq.	Mjarabati (Zaramo), Njitwe (Pare)	T	R	Decoction drunk	LC	[42, 46, 48]

Solanaceae	*Solanum incanum* L.	Ntalantu (Nyamwezi)	H	R, Fr	Decoction drunk	LC	[34]
	*Solanum tuberosum* L.	Ekirazi (Haya)	H	Tu	Peeling then eaten raw	—	[29]
	*Withania somnifera* (L.) Dunal	Olesayiet (Maasai)	S	R, L	Not specified	DD	[44]

Vitaceae	*Cissus rotundifolia* Lam.	Hoza (Zigua), Mchazi (Luguru), Kakulumo (Pogoro)	H	R, L	Decoction drunk	LC	[31, 42, 47]

*Note:* T, tree; H, herb; S, shrub; L, leaf; R, root; B, bark; Se, seed; Fr, fruit; Whp, whole plant; Tu, tuber; VU, vulnerable.

Abbreviations: Ap, aerial parts; CS, conservation status; DD, data deficient; LC, least concern; LF, life form; PU, parts used; Sb, stem bark.

The most frequently cited species include *Sclerocarya birrea* (A. Rich.) Hochst. (Anacardiaceae), *Dichrocephala integrifolia* (L.f.). Kuntze (Asteraceae), *Ageratum conyzoides* L. (Asteraceae), *Dodonaea viscosa* Jacq. (Sapindaceae) and *Cissus rotundifolia* Lam. (Vitaceae), each with three citations (Table 1). These findings demonstrate that the locals regard the aforementioned MPs as the most effective in treating PUD. The noted MPs, *S. birrea* [57], *D. integrifolia* [58], *A. conyzoides* [59], *D. viscosa* [60] and *C. rotundifolia* [61], have been reported in various phytochemical studies to possess antiulcerative activity. Other MPs described elsewhere as having antiulcerative activity include *Allium sativum* L. (Amaryllidaceae) [62, 63], *Psidium guajava* L. (Myrtaceae), *Bidens pilosa* L. (Asteraceae) [64], *Moringa oleifera* Lam. (Moringaceae) [65], *Punica granatum* L. (Lythraceae) [66, 67] and *Zanthoxylum chalybeum* Engl. (Rutaceae) [68]. Therefore, the MPs noted in this review demonstrate a wide array of indigenous knowledge that locals possess regarding the treatment and management of PUD in the country.

### 3.3. Life Forms

**FIGURE 3:**
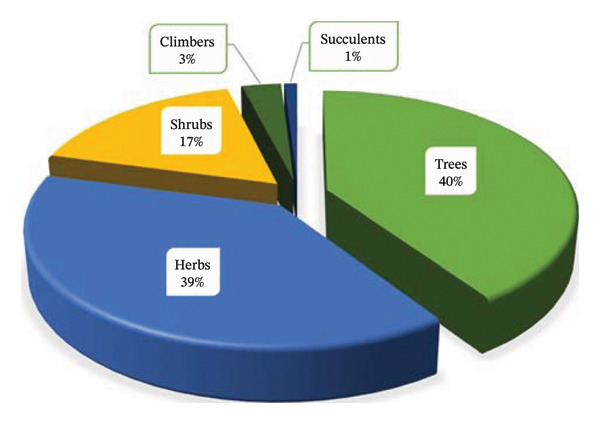
Life forms of medicinal plants used to treat and manage peptic ulcers in Tanzania.

The assessment of the life forms of the recorded MPs used for managing and treating PUD revealed that trees accounted for the largest proportion, with 41 MPs (40%), followed by herbs with 39 MPs (39%), shrubs with 17 MPs (17%), climbers with 3 MPs (3%) and succulent 1 MP (1%) and (Figure 3). Approximately 79% of life forms comprise trees and herbs. This suggests that increasing the use of trees and herbs could be beneficial, as trees are available year‐round, and herbs can be easily cultivated in home gardens, requiring less time to grow. However, herbs may be unavailable during certain seasons, particularly when sourced from the wild [69, 70]. Moreover, several scholars have reported that trees and herbs are the most commonly utilised life forms for managing various human ailments in Tanzania [26, 71, 72] and beyond [52, 73–75].

The observed dominance of trees and herbs in ethnobotanical use carries significant conservation implications, requiring both ecological and institutional responses. Tree‐dominated systems emphasise the importance of forest protection, long‐term habitat stability and carbon sequestration, while herb‐dominated systems rely on sustainable harvesting practices to safeguard species diversity and prevent local depletion. From a governance perspective, these patterns underscore the need for integrated conservation frameworks that align community practices with national policies, thereby ensuring biodiversity preservation, ecological resilience and the sustained availability of medicinal resources. Such alignment not only supports environmental stewardship but also strengthens institutional accountability and continuity in public health and resource management.

### 3.4. Used Plant Parts

**FIGURE 4:**
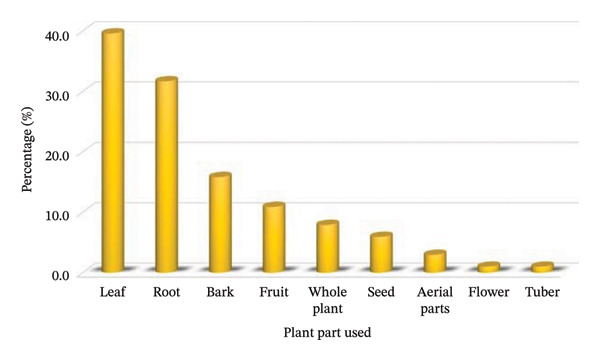
Percentage of plant parts used to treat and manage peptic ulcer disease in Tanzania (note: cumulative percentage exceeds 100% due to overlapping part usages).

In the current review, various plant parts have been identified as effective in the treatment and management of PUD. Leaves are the most commonly utilised part, accounting for 40% of the recorded MPs, followed by roots (32%), bark (16%), fruit (11%), whole plant (8%), aerial parts (3.6%), seed (6%), aerial parts (3%), and flower and tuber, each with 1% (Figure 4). In contrast, fruits, flowers, seeds and aerial parts are seldom used (Table 1). The findings of this review align with ethnobotanical studies conducted within the country [51, 76] and elsewhere, such as Kenya [77], Ethiopia [78, 79] and Uganda [80], which reported leaves and roots as the most frequently utilised plant parts in remedy preparations for various human disorders. The benefit of using leaves is their ease of harvest and year‐round availability. Their use in remedy preparation is generally advocated as a more sustainable practice due to their renewability and lesser detrimental impacts on the parent plant [72, 81, 82]. Unlike leaves, the extensive use of roots can jeopardise the survival of MPs, as their harvest tends to affect anchorage, nitrogen fixation and the root systems of adjacent plants [49, 83].

### 3.5. Preparation and Administration of Remedies

**FIGURE 5:**
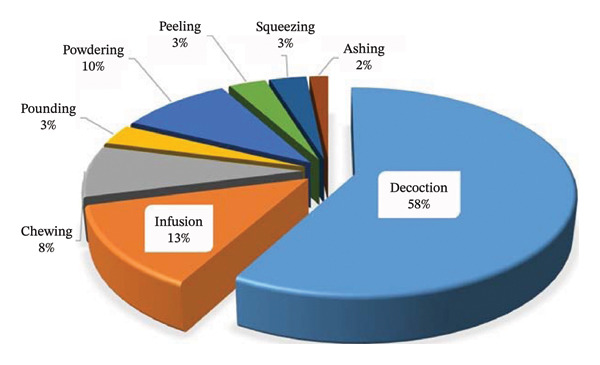
Percentage share of modes of preparation of antiulcer remedies.

Most of the reviewed articles indicate that local traditional practitioners commonly use single or combined parts of the same or different MPs when formulating herbal remedies for the treatment and management of PUD. Some formulations involve adding additives, such as water, honey and milk, to improve flavour and therapeutic potential. The decoction method (58%) remains the primary approach for preparing herbal remedies, alongside other techniques such as infusions (13%), chewing (8%), powdering (10%), pounding (3%), peeling (3%), squeezing (3%) and ashing (2%) (Figure 5). Decoction is often preferred in herbal preparations due to its effectiveness in extracting water‐soluble and heat‐stable compounds from resilient plant parts such as roots, bark and seeds [84, 85]. This method enhances the formation of synergistic compounds, thereby improving the medicinal properties of MPs. It also detoxifies harmful compounds and sterilises the MP materials used [86]. Moreover, the majority of herbal treatments for PUD are administered orally (95.8%), with a small fraction (4.2%) being applied topically (Table 1). This observation aligns with the antiulcer review of preclinical and clinical studies [87], which reported that the oral route is the primary route for administering PUD treatment. Moreover, it is important to note that standardising MP dosages is difficult due to variability in composition, preparation and patient response. This lack of consistency increases the risk of toxicity and calls for rigorous pharmacological evaluation and regulatory oversight.

### 3.6. Conservation Status (CS)

**FIGURE 6:**
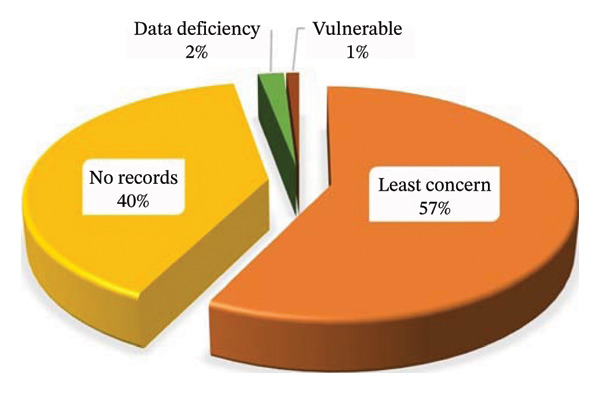
Conservation status of the recorded medicinal plants used to treat peptic ulcers.

The CS of all documented MPs was evaluated using the International Union for Conservation of Nature (IUCN) Red List of Threatened Species (IUCN, 2022). Of the species recorded, 40 MPs were absent from the IUCN database, while 58 MPs were classified as least concern (LC), reflecting stable global populations (Table 1). One MP, *Khaya nyasica* Stapf ex Baker f., was listed as vulnerable, and two MPs, *Withania somnifera* (L.) Dunal and *Carica papaya* L., were categorised as data deficient (Table 1 and Figure 6). From a policy perspective, these results underscore the urgent need to strengthen conservation frameworks to safeguard MPs, particularly those with uncertain or unassessed status, prioritise systematic evaluation of species absent from the IUCN database to inform evidence‐based decision‐making and integrate conservation planning into national and institutional strategies, ensuring that MP resources are sustainably managed for both biodiversity and public health outcomes. Moreover, this study highlights a critical governance gap: without comprehensive conservation assessments, policymakers risk overlooking species that may be vulnerable. Addressing these gaps will require coordinated action between research institutions, conservation agencies and regulatory bodies to ensure long‐term resilience of MP populations.

## 4. Validated Antiulcerogenic MPs

Of the 101 MPs reviewed, only 32 species (31.7%) have been pharmacologically validated for significant antiulcerogenic activity (Table 2), highlighting their therapeutic potential. Several extracts demonstrated exceptional efficacy, with *Bidens pilosa* achieving complete (100%) inhibition of ulcer formation, while *Brassica oleracea*, *Psidium guajava*, *Punica granatum* and *Securidaca longepedunculata* each exceeded 90% inhibition. Other species, such as *Carica papaya* seeds, not only reduced ulcer incidence but also promoted healing in chronic models, emphasising their dual therapeutic roles. These effects are primarily mediated by phytochemicals, including flavonoids, tannins, saponins, terpenoids and distinctive metabolites, such as plumbagin (*Plumbago zeylanica*) [113], monomeric, leucocyanidin (*Musa sapientum*) [109, 123], quercetin, stigmasterol, campesterol (*Psidium guajava*) [114], ellagitannins, punicalagin (*Punica granatum*) [115, 116] and withanolides (*Withania somnifera*) [122]. Collectively, these compounds confer gastroprotection through antioxidant, cytoprotective and anti‐inflammatory mechanisms. Beyond direct mucosal protection, several MPs demonstrated broader benefits; for instance, *Moringa oleifera* enhanced the activities of catalase, superoxide dismutase and glutathione peroxidase [108, 124], while *Aloe vera* restored glutathione levels and produced synergistic effects when combined with *Aegle marmelos* [114]. These findings suggest that antiulcerogenic properties extend beyond ulcer reduction to modulation of oxidative stress and inflammatory pathways, reinforcing the pharmacological relevance of MPs across diverse experimental models.

**TABLE 2:** Pharmacologically validated medicinal plants with antiulcerogenic activity.

Plant	Part used (extract type)	Model used	Key results	Bioactive compounds	Reference
*Ageratum conyzoides*	Leaves (aqueous; ethanolic)	Ethanol‐induced gastric lesion; ibuprofen‐induced ulcer; cold restraint stress‐induced ulcer	Dose‐dependent protection; reduced ulcer index, mean ulcer size and ulcer count; oral administration of ethanol extract (500–750 mg/kg) protected gastric lesions by 80.6% and 89.3%, respectively	Flavonoids, alkaloids	[59, 88]
*Aloe vera*	Leaves (aqueous gel)	Cysteamine‐induced duodenal ulcer; ethanol‐induced gastric ulcer; Indomethacin‐induced gastric ulcer	Strong protection; restored glutathione levels; synergistic pretreatment with *Aloe vera* (150–200 mg/kg) and *Aegle marmelos* significantly inhibited ulcer formation	Anthraquinones, polysaccharides, flavonoids	[89, 90]
*Amaranthus spinosus*	Leaves, whole plant (ethanolic; petroleum ether)	Shay rat (pylorus ligation); ethanol‐induced ulcer; aspirin‐induced ulcer	Lowered ulcer index; protection against ethanol‐induced gastric damage by up to 80.88%	Flavonoids, alkaloids, tannins	[91]
*Annona senegalensis*	Root bark (ethanolic)	Ethanol‐induced gastric ulcer	Marked gastroprotective activity was observed, with a significant reduction in the ulcer index at doses of 100 mg/kg and 200 mg/kg	Alkaloids, flavonoids, saponins	[92]
*Bidens pilosa*	Whole plant (methanolic; methylene chloride)	Ethanol‐induced gastric ulcer; HCl/ethanol‐induced ulcer	Exhibited the strongest antiulcer effect, achieving complete (100%) inhibition; improved mucosal protection	Polyacetylenes, flavonoids (quercetin derivatives)	[93]
*Brassica oleracea var. capitata*	Leaves (aqueous)	Aspirin‐induced gastric ulcer	Inhibited ulcer formation by 99.44%; potent gastroprotection	methionine sulfonium chloride (S‐methylmethionine), flavonoids	[94]
*Carica papaya*	Seeds (methanolic)	Ethanol‐induced ulcer; indomethacin‐induced ulcer; chronic acetic acid‐induced ulcer	Dose‐dependent gastroprotection (up to 90% inhibition); promoted healing in chronic ulcers	Benzyl isothiocyanate, flavonoids, alkaloids	[95, 96]
*Commiphora africana*	Stem bark (methanol; ethyl acetate; hexane)	Ethanol‐induced gastric ulcer	Dose‐dependent antiulcerogenic activity; the methanolic fraction achieves the highest efficacy, reaching up to 90.63% inhibition	Terpenoids, resin acids	[97]
*Crassocephalum vitellinum*	Aerial parts (aqueous; ethanolic)	Ethanol–HCl‐induced ulcer	Dose‐dependent gastroprotection up to 88.3% at 800 mg/kg; reduced ulceration	Flavonoids, terpenoids, tannins, saponins	[98]
*Cucumis sativus*	Seeds (methanolic)	Ethanol‐induced ulcer in rats	The extract reduced gastric acid volume, free acidity and total acidity by 41%, 48% and 29% at 300 mg/kg. Ulcer index inhibition was 52.5% (PL) and 62.7% (WIS) at the higher dose	Flavonoids, tannins, saponins	[99]
*Cucurbita pepo*	Seed (methanolic)	Pylorus ligation and ethanol‐induced ulcers	Demonstrated a significant decrease in ulcer index, with inhibition levels recorded between 55.7% and 67.1%.	Cucurbitane‐type triterpenoids	[100]
*Daucus carota*	Roots (aqueous)	Ethanol‐induced ulcer in rats	Marked reduction of gastric lesions with improved mucosal protection at 500 mg/kg	Carotenoids, flavonoids	[101]
*Dodonaea viscosa*	Leaves (ethanolic)	Ethanol‐induced ulcer; indomethacin‐induced ulcer	Marked reduction of ulcer index in both models by 52.8% and 54.9%.	Flavonoids, saponins, tannins, sterols	[102]
*Emilia coccinea*	Leaves (aqueous)	Ethanol‐induced gastric ulcer	Demonstrated notable antiulcerogenic activity at 400 mg/kg, lowering the ulcer index and oxidative stress	Flavonoids, phenolic acids	[103]
*Jatropha curcas*	Leaf (methanolic)	Aspirin‐induced ulcers in rats	Dose‐dependent reduction in ulcer index (100–200 mg/kg) with cytoprotective effect	Flavonoids, alkaloids, saponins	[104]
*Kigelia africana*	Stem bark (ethanolic)	Aspirin‐induced ulcer	At a dose of 450 mg/kg, the extract produced the most pronounced effect, with a significant reduction in ulcer index	Saponins, tannins, phylobatannins, anthraquinones, cardiac glycosides	[105]
*Leonotis nepetifolia*	Leaf extract	Pylorus ligation and ethanol‐induced ulcers	Reduced gastric volume, ulcer index as well as an elevation in gastric juice pH	Flavonoids, terpenoids	[106]
*Maytenus senegalensis*	Root bark (methanolic, hydroethanolic)	Ethanol‐induced gastric ulcer in Wistar rats	Significant reduction in ulcer index, dose‐dependent gastroprotection and improved mucosal defence	Triterpenoids (maytenin, pristimerin), triterpenoids (maytenin, pristimerin), flavonoids, alkaloids	[107]
*Moringa oleifera*	Pods, roots (hydroalcoholic; methanolic)	Indomethacin‐induced ulcer; ethanol‐induced ulcer	Reduced ulceration by 79%; lowered MDA; enhanced antioxidant enzymes (CAT, SOD, GPx) activities	Flavonoids, phenols, tannins	[108]
*Musa sapientum*	Fruit pulp (Aqueous)	Indomethacin‐induced ulcer	At a dose of 100 mg/kg/day, the extract strengthened the gastric mucosa by stimulating cell proliferation, enhancing mucus secretion and resistance, suppressing HCl secretion and promoting ulcer healing	Monomeric, leucocyanidin	[109]
*Persea americana*	Leaf (aqueous)	Ethanol and indomethacin‐induced ulcers	Demonstrated a significant reduction in ulcer index with suppressed gastric acid secretion	Flavonoids, phenolics	[110]
*Phyllanthus amarus*	Leaves (aqueous; acetone)	Ethanol‐induced ulcer	Gastroprotective and antioxidant effects; reduced ulceration by 41.2%–59.3%	Lignans (phyllanthin, hypophyllanthin)	[111]
*Piliostigma thonningii*	Leaf (hydromethanolic)	Ethanol‐induced ulcers in rats	The extract demonstrated a reduction in ulcer index with a cytoprotective effect at 100 mg/kg	Flavonoids, tannins, saponins	[112]
*Plumbago zeylanica*	Roots (methanolic)	Preclinical gastric ulcer model	Exhibited anti‐inflammatory and cytoprotective effects, with clear evidence of ulcer protection	Plumbagin	[113]
*Psidium guajava*	Leaves, bark, seeds (ethanolic)	Ethanol‐induced ulcer	Demonstrated strong antiulcer activity, with gastroprotective potential reaching a preventive index of 98.6%, and enhanced mucosal defence	Quercetin, tannins, stigmasterol, campesterol	[114]
*Punica granatum*	Peel (methanol; hydroalcoholic)	Ethanol‐induced ulcer; indomethacin‐induced ulcer	Exhibited strong antiulcerogenic activity, reaching up to 91.5%	Ellagitannins, punicalagin, ellagic acid, phenolics, flavonoids	[115, 116]
*Securidaca longepedunculata*	Root (methanol, ethylacetate, hexane)	Ethanol‐induced ulcers in rats	Strong antiulcer activity with inhibition of 68.8%–93.8%	Xanthones, saponins, flavonoids	[117]
*Solanum incanum*	Leaves, roots (hydromethanol)	Ethanol‐induced ulcer	Reduced ulcer index after 10 days (100–400 mg/kg), showing pronounced cytoprotective activity and fewer gastric lesions	Phenols, flavonoids, saponins	[118]
*Solanum tuberosum*	Tuber (methanolic)	Ethanol‐induced ulcers in rats	The extract reduced ulcer index and provided mucosal protection	Polysaccharides, phenolics	[119]
*Sonchus oleraceus*	Aerial parts, roots (alcoholic)	Ethanol‐induced gastric ulcer; ulcerative colitis	The extract at a dose of 500 mg/kg showed strong antiulcerative colitis activity with 88.5% protective index	Sesquiterpene lactones, flavonoids	[120]
*Syzygium cumini*	Seeds (methanol; ethyl acetate)	Ethanol‐induced ulcer; indomethacin‐induced ulcer	Demonstrated significant antiulcer activity at the dose level of 200 mg/kg, with enhanced mucosal cytoprotection	Flavonoids, tannins	[121]
*Withania somnifera*	Roots (aqueous; hydroalcoholic)	Stress‐induced ulcer; ethanol‐induced ulcer	Gastroprotective effects; reduced ulceration in stress and ethanol paradigms	Withanolides	[122]

Despite substantial preclinical evidence of antiulcerogenic effects, translating these findings into clinical practice requires rigorous validation [87, 125]. To fully realise their therapeutic potential, future research should prioritise well‐designed clinical trials, standardised formulations, mechanistic insights and comprehensive safety assessments to ensure treatment reliability. Importantly, traditionally used but scientifically unvalidated MPs should be systematically examined to ensure that culturally rooted remedies are evidence‐based and safely incorporated into modern healthcare. Validated MPs offer not only therapeutic opportunities but also strategic resources for advancing Sustainable Development Goal 3 (SDG3) (Good Health and Well‐being), through their evidence‐based integration into national health systems and the safe inclusion of traditional medicine within modern treatment frameworks.

## 5. Need for Conservation of MPs

The conservation of MPs is essential for maintaining biodiversity, ensuring sustainable healthcare and preserving traditional knowledge. Protecting these valuable plants requires collective efforts by local communities and policymakers. Sustainable practices, reforestation and habitat conservation are crucial for their protection [126, 127]. Some key reasons why MPs should be safeguarded include the following.

### 5.1. Biodiversity and Ecosystem Balance

MPs are essential components of ecosystems, supporting wildlife and maintaining ecological balance. The decline or loss of these plants can disrupt food chains and degrade natural habitats, thereby affecting the overall health of the environment [128]. Therefore, traditional health practitioners and conservation authorities in the country play an essential role in safeguarding MPs and their associated ecosystems, thereby ensuring the sustainable availability of resources for herbal remedy formulations.

### 5.2. Sustainable Healthcare and Medicine

A significant number of contemporary pharmaceuticals stem from the intricate chemistry of plant‐derived compounds. Conserving these MPs ensures a vital, sustainable supply of natural resources essential for advancing research and drug development [129]. This practice not only preserves the esteemed heritage of traditional medicine but also encourages innovation in modern healthcare, ultimately benefiting society as a whole.

### 5.3. Cultural and Indigenous Knowledge

Since time immemorial, indigenous communities have utilised traditional medicine, primarily relying on MPs. Protecting these practices is vital, as it preserves both essential botanical knowledge and cultural heritage. Safeguarding traditional medicine ensures that critical insights into natural healing are maintained for future generations [130, 131].

### 5.4. Economic Benefits

MPs play a significant role in strengthening the economy, serving as essential resources for the pharmaceutical industry, enhancing the production of herbal remedies and facilitating local trade networks. By utilising their therapeutic properties, these plants not only advance health but also contribute to substantial economic activity. Moreover, the conservation of these invaluable resources is imperative to ensure long‐term economic sustainability, allowing future generations to benefit from their medicinal properties while safeguarding the rich biodiversity within our ecosystems [132–134].

### 5.5. Climate Resilience

Numerous MPs exhibit remarkable adaptability to challenging climatic conditions, playing a vital role in maintaining soil health. Thus, preserving these plants is crucial, as it enhances ecosystem resilience to climate change [135].

### 5.6. Prevention of Overharvesting and Extinction

The unregulated collection of MPs, coupled with the rampant destruction of their habitats, poses a significant threat to biodiversity. Implementing robust conservation programmes is vital for promoting responsible harvesting practices and ensuring the sustainable utilisation of these invaluable resources [136]. Thus, collectively, natural heritage can be safeguarded for future generations.

Thus, it is imperative to recognise that conserving MPs necessitates a comprehensive strategy to ensure their long‐term sustainability and availability for future generations. Key strategies include, but are not limited to, *in situ* conservation, *ex situ* conservation, sustainable harvesting practices, cultivation and domestication, robust policy and legal frameworks, active local community engagement and provision of conservation awareness and rigorous scientific research on MPs properties, propagation techniques and genetic conservation, which enhances their sustainable use and medicinal potential [81, 137–139]. By integrating these strategies, the challenges facing MPs can be effectively addressed, hence ensuring their long‐term availability.

## 6. Future Perspective on MPs for PUD

The therapeutic potential of MPs in PUD is closely linked to their diverse pathophysiological mechanisms, including enhancement of gastric mucosal defence, stimulation of mucus and bicarbonate secretion, free radical scavenging, modulation of inflammatory pathways, inhibition of gastric acid secretion and promotion of angiogenesis and tissue regeneration. These multifaceted actions highlight how MPs directly target the underlying processes of PUD, offering a holistic approach compared with single‐target synthetic drugs [87]. Building on these mechanisms, research into MPs holds considerable promise for advancing healthcare and pharmacology. As global interest in natural and traditional medicine escalates, studies on plant‐based remedies for ulcers are expected to expand, culminating in the identification of more effective, accessible and sustainable therapeutic alternatives. Future investigations should prioritise isolating bioactive compounds with potent gastroprotective properties [140], while innovations in biotechnology and nanotechnology are anticipated to enhance efficacy and bioavailability, thereby improving drug formulations. With clinical trials confirming effectiveness, MPs may achieve broader acceptance within mainstream medical treatments [87, 141]. Realising this potential requires sustainable harvesting and cultivation practices to ensure continued availability, alongside conservation initiatives such as genetic mapping and tissue culture to safeguard biodiversity [127, 142]. Commercialisation of herbal antiulcer therapies is likely to grow, supported by investment and evolving regulatory frameworks that promote safe, standardised use [143]. Although conducted in Tanzania, this review contributes to the global evidence base on MPs for PUD. Documenting ethnomedicinal practices and biodiversity unique to Tanzania provides novel bioactive leads that can inform international drug discovery and therapeutic development [29]. Such locally grounded research underscores the importance of integrating indigenous knowledge into modern pharmacology, offering models for sustainable and culturally relevant healthcare worldwide [144]. Consequently, findings from Tanzania hold significance for shaping global strategies in PUD management and herbal medicine regulation.

## 7. Specific Regulatory Challenges to Tanzania

The regulation of herbal remedies in Tanzania remains a complex challenge, shaped by gaps in enforcement, limited laboratory infrastructure and inadequate clinical validation. Although the Traditional and Alternative Medicine Act (2002) provides a legal framework for oversight, weak institutional capacity and resource constraints hinder effective monitoring by agencies such as the Tanzania Food and Drugs Authority and the National Institute for Medical Research. Many traditional healers continue to operate outside formal registration systems, reflecting low practitioner compliance and mistrust of biomedical institutions [145]. Ethical review processes for herbal clinical trials are further complicated by insufficient expertise in ethnobotany, which leads to delays and inconsistencies in approvals [146]. These regulatory shortcomings pose risks to patient safety, undermine public trust and limit the global recognition of Tanzanian herbal remedies. Addressing these challenges requires developing a national herbal pharmacopoeia, investing in laboratory infrastructure, enhancing practitioner training and strengthening collaboration between biomedical researchers and traditional medicine practitioners to ensure the safe, evidence‐based integration of herbal remedies into the national healthcare system.

## 8. MPs and Sustainable Development Agenda

From a sustainability perspective, the findings of this review align closely with several United Nations SDGs. MPs contribute to SDG 3 (Good Health and Well‐being) by offering affordable, accessible and culturally relevant treatments for ulcer management, thereby reducing reliance on costly synthetic drugs. Their cultivation and responsible use foster SDG 15 (Life on Land) by conserving biodiversity and protecting traditional ecological knowledge. Integrating validated therapies into formal healthcare systems advances SDG 9 (Industry, Innovation and Infrastructure) by bridging traditional medicine and modern innovation and strengthening health delivery frameworks. Equally, the development of standardised, sustainable production practices supports SDG 12 (Responsible Consumption and Production) by ensuring quality, safety and equitable resource use. Taken together, these contributions underscore the dual relevance of MPs to pharmacology and sustainability, positioning them as both therapeutic agents and drivers of sustainable development.

## 9. Conclusion

The use of 101 MPs in managing PUD in Tanzania reflects a rich tradition of ethnomedical knowledge that continues to offer promising therapeutic options. Plants such as *Sclerocarya birrea, Dissotis integrifolia, Ageratum conyzoides, Dodonaea viscosa* and *Cissus rotundifolia* are notable for their high citation counts. Of these recorded MPs, 32 have been pharmacologically validated for antiulcerogenic activity, with several demonstrating inhibition capacities exceeding 90%. These findings not only support traditional practices but also highlight the potential to develop affordable, accessible and culturally acceptable remedies. Ongoing scientific research and clinical validation are essential to fully harness the benefits of these natural therapies and incorporate them into modern healthcare systems. Likewise important, MPs that have not yet been validated for antiulcerogenic activity should be prioritised for detailed phytochemical, pharmacological and clinical studies to evaluate their safety, efficacy and mechanisms of action. This balanced approach ensures that promising candidates are recognised and that traditional knowledge is systematically translated into evidence‐based practice. This review also emphasises the importance of conserving MPs and increasing local awareness of cultivating these plants in botanical or home gardens to secure their preservation for future generations. Additionally, documenting these practices provides a foundation for comparative global research on MPs for PUD, fostering cross‐cultural collaboration and expanding the evidence base for integrating ethnomedicine into international health frameworks.

## Author Contributions

D.S.K. designed the study, analysed data, drafted the manuscript and approved the final submission.

## Funding

This review received no funding.

## Ethics Statement

As this was a systematic review, ethical approval and consent to participate were not required.

## Conflicts of Interest

The author declares no conflicts of interest.

## Data Availability

The information supporting the findings of this study can be obtained from the corresponding author upon request.
